# Supplementary material for: The effectiveness and characteristics of mHealth interventions to increase adolescent’s use of Sexual and Reproductive Health services in Sub-Saharan Africa: A systematic review
Source: PLoS One. 2022 Jan 21;17(1):e0261973. doi: 10.1371/journal.pone.0261973 (PMC8782484; doi:10.1371/journal.pone.0261973)
Supplement: S1 File — (DOCX) [file pone.0261973.s002.docx]

**Supplementary file**

**Literature search strategy**

Search terms were iteratively developed within each of three search concepts: (i) Sexual and reproductive health; (ii) mHealth; (iii) Sub-Saharan Africa. The keywords and database thesaurus terms were combined using Boolean Operators and truncation/wildcards were applied and modified where appropriate. Details of the search strategy are included as supplementary fill and the full review protocol is published online. For example, the preliminary search strategy in MEDLINE using the key concepts are shown below.

Concept 1 - Sexual and reproductive health

Reproductive health [MeSH] OR sexual health [MESH] OR reproductive health services [MESH] OR Sexuality [MESH] OR Maternal Health services [MESH] OR SRH or SRHR OR ASRH OR contracept* OR family W2 planning OR “Family planning service*” OR condom or Birth W2 control OR pregnan* OR abortion OR pregnancy W2 termination OR unprotected W2 Sex OR sexually W2 transmitted OR transmitted W2 disease* OR STI* OR STD* OR Human W2 Immunodeficiency OR Acquired W2 immune-deficiency OR HIV OR AIDS OR HIV W2 Testing OR HIV W2 Infection* OR Sexual W2 activit* OR sexual W2 intercourse OR antenatal W2 Care OR prenatal W2 deliver* OR Prenatal W2 Care OR childbirth OR maternal W2 health OR Antiretroviral W2 Treatment OR HIV W2 Care OR AID* W2 Care OR medication W2 Adherence OR “reproductive tract infection*”

Concept 2- mHealth

mHealth OR telemedicine [MeSH] OR Cell phone [MESH] OR “tablet computer”* [MeSH] OR m-health OR mobile health OR eHealth OR e-health OR Telephone OR mobile W2 app OR mobile W2 application OR smart* phone W2 app* OR phone OR SMS OR mCare OR short W3 messag* system* OR Text W3 messag* OR telehealth OR multi* W2 media OR Digital W2 assistant* OR PDA OR pod*cast OR teleconsult* OR telehealthcare OR health W2 messag* OR wireless W2 technology OR Computer-Based W2 Counsel* OR User-Computer W2 Interface* OR “Cell Phone Use”

Concept 3-Sub-Saharan Africa (Based on World bank classification)

Sub-Saharan Africa* OR SSA OR Africa South of the Sahara [MeSH] OR Africa* OR “Low and middle income countr*” OR LMIC OR Low-income W2 countr* OR “third world country” OR “Third world nation*” OR Developing W2 nation* OR Developing W2 countr* OR “least developed countr*” OR underdeveloped W2 countr* OR remote W2 region* OR Angola* OR Ethiopia* OR Niger* or Benin* or Gabon* or Nigeria* or Botswana* or Gambia* or Rwanda* or Burkina Faso* or Ghana* OR Ghanaian* OR Burundi* OR Guinea* OR Senegal* OR Cape Verde* OR Guinea-Bissau* OR Seychelles* OR Cameroon* OR Kenya* OR Sierra Leone* OR Central African Republic* OR Lesotho*OR Somalia* OR Chad OR Liberia* OR South Africa* OR Comoros OR Madagascar* OR South Sudan* OR Congo* OR DRC OR Malawi* OR Sudan* OR Congo* OR DRC* OR Mali* OR Tanzania* OR Côte d'Ivoire OR Mauritania* OR Togo* OR Equatorial Guinea* OR Mauritius* OR Uganda* OR Eritrea* OR Mozambique* OR Zambia* OR Eswatini* OR Namibia* OR Zimbabwe*.
