# Supplementary material for: The effectiveness and characteristics of mHealth interventions to increase adolescent’s use of Sexual and Reproductive Health services in Sub-Saharan Africa: A systematic review
Source: PLoS One. 2022 Jan 21;17(1):e0261973. doi: 10.1371/journal.pone.0261973 (PMC8782484; doi:10.1371/journal.pone.0261973)
Supplement: Attachment — Submitted filename: Response to reviewers.docx [file pone.0261973.s003.docx]

| Sections | **Reviewers comment** | **Responses** |
| --- | --- | --- |
| Overall summary | This review examines the evidence for mHealth interventions to increase adolescent’s use of sexual and reproductive health services in sub-Saharan Africa. The authors conclude that mHealth interventions are effective and cost-effective; however, my interpretation of the results is that they were mixed, and it is not so clear that they were effective or cost-effective overall | We agree with the reviewer's comment. On further scrutiny of the results, we realise we were over-optimistic about the results overall. The results are well captured in the main body of the manuscript, with nuanced findings reported, but in the abstract, the overall summary did not capture the uncertainty. The phrase about cost-effectiveness has been removed as none of the studies measured this outcome. Scanty data were provided on costs.  See lines 35-41. |
| Introduction | Clear definition of mHealth. A definition of sexual and reproductive health would also be helpful here. | We have clearly defined sexual and reproductive health and clarified what we meant by sexual and reproductive health services in the context of our study. Lines 68-73. |
|  | The authors state that sub-Saharan Africa “had the highest prevalence of adolescent pregnancy in the world” (line 48) but do not state which year or when they are referring to. | We have specified the year. See lines 54-56. |
|  | It would be helpful to know the coverage of mobile phone subscribers (line 51 mentions 600 million mobile phone subscribers, but do we know how many will not have access to mobile phones?). | We have given an indication on this. See lines 57-58 |
|  | Although the aim seems clear, it is a little unclear whether the authors were also intending to review the evidence for improving sexual and reproductive health in addition to service use (objective ii) as these are not necessarily the same thing. | Our intention was to include evidence of improving Sexual and Reproductive Health, even though most studies reviewed focused on service use. |
| Method  Inclusion/exclusion criteria  Bias assessment | A clear definition of the population would be helpful here (for example, the age range considered to be adolescent), possibly in a PICO format. This could also expand on the inclusion criteria (for example, study design and outcomes) | The population of interest has been clearly specified and added to the manuscript. Also, the inclusion criteria. See page 6, lines 119-121. |
|  | No indication of restrictions by publication year or language – I note this is mentioned in the protocol, but it would be helpful to know if this was followed and whether any non-English language papers were identified, and if so, who translated those manuscripts. | A statement has been added to the paper indicating there is no restriction in terms of language or publication year. Non-English language published papers on mHealth in SSA were not identified during our literature search. Lines 138-140. |
|  | Lines 146-147 state that “statistical pooling is not possible in this study” but a few words explaining why would be helpful for readers | A explanation on why statistical pooling was not possible has been added - Line 176-179 |
|  | The supplementary search strategy describes the MEDLINE search strategy that is provided as the “preliminary search strategy” – was this the final search strategy used for MEDLINE? How were the three concepts combined? | Yes, this was the final search strategy used for MEDLINE. We combined the concepts one after the other. i.e. concept 1 + concept 2 + concept 3 using Boolean operators (AND and OR). Lines 144-146. |
|  | First, the inclusion and exclusion criteria used in this study are not entirely clear. I will suggest the authors explicitly state this in the paper. | This has been explicitly stated in the paper. Although this was originally defined in the protocol. Lines 122-129, and 138-140. |
|  | That the evidence on which the bias assessment is carried out are not explicit or systematic enough in the text, and that in some cases the limitations of the included studies are not explicit enough.  There is limited information about risk of bias. Without this, it is difficult to interpret the results. A table or similar with the full risk of bias assessment for each paper would be helpful in interpreting the strength of the evidence. Similarly, I could not identify information regarding uptake of the intervention in control/intervention groups and whether intention to treat analyses were undertaken, which would again affect interpretation. | Detail information about the risk of bias provided under the section ‘Characteristics of studies. We clarified and provided information on the random sequence generation, method of concealment, losses to follow, selection of participants, measurement of interventions, departures from intended interventions, and confounding factors for the reported studies, which I suppose provides clarity on the quality of the included studies.  See pages 9 & 10, lines 194-208. |
| Results | Table 1 describes the sample description. There does not seem to be a definition of adolescents, but I note the age ranges of the included papers vary widely and seem to include older adults too (e.g. Harrington et al is described as 14 years and above so does that include all adults?). Clarification is needed of the population included. As a minor point, there is a need to ensure consistency in the formatting of Table 1. | On page 5, lines 100-101, we did specify the age group of the study participants. However, we have expanded on it. Lines 108-111.  Also, we stated in the study protocol that the target population is adolescents defined as any person between ages 10 and 19 years (WHO, 2014). However, we did mention that interventions that focus on young people aged 10-24 years will be considered given that the interventions that focus on young people aged 10-24 may likely not be different from those of adolescents aged 10-19 years. Lines 119-121  Regarding the study by Harrington et al, the target population was women aged 14 years or above. We were unable to disaggregate the data for young women aged 14-24 years. We have acknowledged this as a limitation. See lines 463 to 468.  Table 1 formatted correctly. |
|  | It is good to see a PRISMA flow diagram, but it is not clear from this where the 132 records were identified from that were not from database searching, and whether the 10 included studies were identified from database searching or by other means which would be helpful to know. | The 132 records were studies identified through other repositories such  ProQuest, International Bibliography of social sciences, OpenDOAR, Ethos-British Library, Network digital library of Thesis and Dissertation and ZETOC as specified in the paper. Lines 135-137.  All the 10 studies mainly came from the primary databases such as MEDLINE, CINAHL, PsycINFO etc. |
|  | Figure 3 is helpful to show how the studies adopted the mERA essential criteria. It could be helpful to show Figure 3 by individual studies, so that the reader is able to see how well individual studies conformed to mERA criteria, given the different outcomes included in each study and the heterogeneity of studies overall. | Figure 3 has been modified to highlight the individual studies that make up each component. A footnote has been provided in fig 3 to further illustrate this and the section in this paper ellobaroated. Lines 342-351. |
|  | For the pregnancy and childbirth section, I note odds of self-reported pregnancy are given (line 270) but it is unclear to me whether this is unintended pregnancy or intended pregnancy or intention unknown? | Good observation. However, the intention was not specified by the author of the paper |
|  | For the cost-effectiveness section (line 358), it would be helpful to describe how strong the evidence was for cost-effectiveness. A marginal cost is reported, but again it would be helpful to see what outcome this was referring to. Given that most studies did not report cost-effectiveness, it seems that this systematic review cannot interpret much in terms of the cost effectiveness of mHealth interventions in SRH overall. | We agree that few studies evaluated the cost-effectiveness and costs of intervention development and delivery. We have modified the statement about cost-effectiveness taking into account strength and the body of evidence and adding detail about the outcomes. |
| Discussion | Well written discussion overall.  Potential harms of mHealth are not discussed in any detail, yet one study showed increased sex without a condom in the intervention arm so this could be a risk. | We agree that mHealth could pose potential harms. However, it was only one study out of 10 that reported on the negative association which maybe an artefact. A statement has been provided to clarify this. See page 22, lines 414-419. |
|  | The fact that non-randomised studies did not report controlling for confounding or accounting for missing data (lines 169-170) seems to me to be a significant issue and perhaps should be mentioned in the discussion in more detail as this will have substantial implications for interpretation of the results of those studies. | This has been acknowledged as a limitation in the study. See page 466-468. |
|  | In terms of the mERA checklist, the authors note that completeness of reporting is poor. It seems concerning that only 2 included information on infrastructure, and only 1 included access of individual participants in terms of barriers and facilitators to the adoption of the intervention among study participants. This seems a considerable limitation of included studies and it is surprising not to see this discussed in more detail in the discussion. | This section has been expanded to capture the limited reporting of this important features, and the reason why this might be this case 433-441 |
|  | Secondly, the discussion section could be generally improved. The discussion is an opportunity to provide explanations of the results and the possible implications. There is a need to provide a better explanation of how some of the conclusions stated in this section were reached. For example, four out of 10 studies reported acceptability, three reported feasibility, and two reported cost-effectiveness. However, you stated in line 396 that “Finally, the results of our review showed that mHealth interventions were cost-effective, acceptable and can feasibly be delivered among adolescents in SSA.” Are the findings strong enough to support this assertion? If so, please explain. | We agree that the statement about feasibility, acceptability and cost effectiveness are too strong given that few studies evaluated these outcomes. We have modified these statements to provide a more nuanced interpretation, relating the findings to the types of intervention aims and study population. |
| Minor comments | Minor issues:  I suggest the heading “mHealth platforms used” be re-titled as “mHealth interventions and platforms used.” Still, under this section heading, you mentioned eight studies made use of SMS. However, it is not clear what mHealth platforms the other two studies used. It might be good to account for all the reviewed studies in the explanation.  The first secondary objective states, “Acceptability of mHealth interventions to adolescents, and parents in providing SRH information in SSA.” However, when reporting the findings for this objective, there was no mention of parents. It might be good to consider this when discussing your findings here.  Line 43 – “…young people face enormous barriers accessing Sexual and Reproductive Health (SRH) information and services.” Would you please highlight some of these barriers? | Good suggestion. The title has been corrected.  A statement has been added to clarify other platforms used. See lines 212-213  A sentence has been added to indicate that none of the studies reported on the acceptability of the mHealth platform by parents of adolescents. See lines 363-364.  We have clarified this. See lines 49-51 |
